# Methodologies for studying depression in persons with tuberculosis: A scoping review

**DOI:** 10.1371/journal.pgph.0007388

**Published:** 2026-09-28

**Authors:** Amanda J. Gupta, Patricia Turimumahoro, Fulera Salami, Ayden Brennan, David Jacoby, Sheeba Ibidunni, Lori Rosman, Jonathan E. Golub, David W. Dowdy

**Affiliations:** 1 Johns Hopkins Bloomberg School of Public Health, Baltimore, Maryland, United States of America; 2 World Alliance for Lung and Intensive Care Medicine in Uganda (WALIMU), Kampala, Uganda; 3 Epidemiology of Microbial Diseases, Yale School of Public Health, New Haven, Connecticut, United States of America; 4 Welch Medical Library, School of Medicine, Johns Hopkins University, Baltimore, Maryland, United States of America; 5 Department of Epidemiology, Johns Hopkins Bloomberg School of Public Health, Baltimore, Maryland, United States of America; 6 Department of International Health, Johns Hopkins Bloomberg School of Public Health, Baltimore, Maryland, United States of America; 7 Center for Tuberculosis Research, Johns Hopkins University School of Medicine, Baltimore, Maryland, United States of America; PLOS: Public Library of Science, UNITED STATES OF AMERICA

## Abstract

Depression is prevalent among people with tuberculosis (PWTB) and negatively affects diagnosis, treatment adherence, and clinical outcomes. However, the methodological approaches used to study depression among PWTB have not been systematically mapped. This scoping review aimed to evaluate methodologies used to study depression among PWTB and identify gaps in methodological approaches. We conducted a systematic scoping review following PRISMA-ScR guidelines. Nine databases were searched without date restrictions. Two reviewers independently screened titles, abstracts, and full texts. Inclusion criteria included primary, peer-reviewed, research studies that examined depression among PWTB, and reported outcomes related to TB and depression. Data were extracted on study characteristics, TB population, depression measurement tools, treatment approaches, and implementation considerations. Findings were organized using Reach, Effectiveness, Adoption, Implementation, Maintenance (RE-AIM) framework. A total of 184 studies met inclusion criteria. Research was dominated by cross-sectional designs (65%), with fewer cohort studies (17%) and only eight randomized controlled trials (RCTs, 4%). The RCTs primarily evaluated psychological interventions that demonstrated modest improvements in depressive symptoms or TB-related outcomes. Depression was overwhelmingly assessed using screening tools with only 28 studies (15%) using a diagnostic interview or clinical criteria. None evaluated accuracy of any screening or diagnostic tool. Studies were geographically clustered, with more than one-third conducted in India or China. Implementation barriers to integrated TB and depression care included workforce shortages and stigma; facilitators included task-shifting and embedding screening into routine workflows. No studies reported on maintenance or long-term sustainability of depression care within TB programs. Existing research on depression among PWTB is largely cross-sectional, relies heavily on screening tools, and rarely evaluates interventions or their sustainability. Expanding population and geographic coverage, incorporating longitudinal designs and diagnostic evaluation, and strengthening implementation and maintenance research are critical for developing sustainable, integrated approaches to depression care within TB services.

## Introduction

Despite decades of research and public health eradication efforts, tuberculosis (TB) stubbornly remains a leading cause of morbidity and mortality. In 2024 alone, 10.7 million people fell ill with TB and 1.23 million died from the disease [1]. Efforts to end TB have been stymied by persistent gaps in diagnosis and treatment, and a lack of integration of care for comorbid conditions, especially in high TB burden settings [2–4]. Comorbidities are especially important in TB care as they are pervasive amongst persons with TB (PWTB) and are often associated with negative treatment outcomes such as poor adherence, death, or loss to follow-up [4–7].

One such co-morbidity is depression, a common mental health condition marked by depressed mood and reduced interest or pleasure in usual activities, that may be more prevalent among PWTB than the general population [8]. While estimates vary, systematic reviews have estimated that between 30–45% of PWTB have comorbid depression [9–11]. Depression can delay TB diagnosis, reduce treatment adherence, and worsen clinical outcomes [12–14]. The relationship between depression and TB may be bidirectional, as TB can exacerbate depressive symptoms through social isolation, stigma, and biological stress responses while depression may increase susceptibility to TB through immune dysregulation associated with chronic psychological stress [15]. Overlapping symptomatology, such as fatigue, appetite loss, or sleep disturbance, between depression and TB further complicates the relationship leading to measurement errors and under-treatment in practice.

Although this complex interplay is largely recognized, most research to date has narrowly focused on establishing the prevalence of depression amongst PWTB. Few studies have employed longitudinal designs to capture the detailed patient journey of those experiencing both TB and depression. Even fewer have employed experimental designs to evaluate the impact of interventions on outcomes for either condition. A systematic synthesis of methodology is therefore necessary to identify existing gaps and guide more rigorous, integrated research and public health efforts.

To address this gap, we conducted a systematic scoping review to characterize how depression has been studied among PWTB, examining study designs, diagnostic approaches, and treatment methodologies, and to identify priority areas for future research.

## Methods

### Ethics statement

This research was determined to be non-human subjects research by the Johns Hopkins Bloomberg School of Public Health Institutional Review Board as all data utilized were publicly available and previously published. Because no human participants were involved and no identifiable private information was accessed, institutional review board approval and informed consent were not required.

### Reporting guidelines and protocol

This review was conducted in accordance with the framework for scoping reviews proposed by Levac et al [16] with the full protocol previously published [17]. This review is reported according to Preferred Reporting Items for Systematic Reviews and Meta-Analyses extension for Scoping Reviews (PRISMA-ScR) [18].

### Research question

The question for our review was: what methodologies have been used in the research literature to study depression (including diagnosis and treatment) among PWTB, and what are the gaps in methodological approaches?

### Eligibility criteria and search

The full eligibility criteria are listed in Table 1. In short, we included primary, peer-reviewed, research studies that examined depression among PWTB, encompassing all TB types, and that reported outcomes related to the intersection of TB and depression. We excluded studies that solely focused on TB or depression without examining the two conditions together, were conducted in non-human or laboratory settings, or were non-empirical, as well as review articles and studies not available in English. To capture the full scope of existing evidence, we did not restrict the search by publication date.

**Table 1:** Study eligibility criteria.

Domain	Included	Excluded
Participants	PWTB regardless of age, sex, socioeconomic status and TB type including: • Microbiologically confirmed • Clinically diagnosed • Pulmonary • Extra-pulmonary • Latent • Active • Drug-resistant • Drug-sensitive	• Studies involving only those without a confirmed TB diagnosis • Studies with animal participants • Participants with nontuberculous mycobacterial infections (e.g., leprosy) • Studies that only include laboratory samples and isolates
Concept or intervention	Studies that contain information on depression and tuberculosis including: • Diagnostic tools and methods • Therapeutic interventions (pharmacological or psychological) • Determinants of the TB-depression syndemic and underlying pathways and mechanisms	• Studies that explore tuberculosis or depression separately • Studies that only focus on biological mechanisms of tuberculosis and/or depression
Outcome	• Sensitivity, specificity, and/or accuracy of depression diagnostic tools in PWTB • Studies reporting diagnostic challenges, barriers, and/or facilitators • Validation of depression diagnostic measures in PWTB • Changes in depression symptoms (including patient-reported outcomes) during TB treatment • TB treatment adherence, completion and/or complication rates related to depression • Studies with outcomes on methodological challenges in studying depression and tuberculosis concurrently	Outcomes not explicitly linked to the study of depression in PWTB such as: • Studies reporting biochemical, genetic, or molecular outcomes without clinical or diagnostic relevance • Studies with general TB treatment outcomes unrelated to or not considering depression • Studies with insufficient detail on how outcomes were measured or analyzed • Studies based solely on opinions or informal observations
Context	Research conducted in healthcare or community settings of relevance Global geographic inclusion	Research from unrelated contexts such as veterinary or laboratory studies
Study Design	• Primary studies • Randomized controlled trials • Cross-sectional studies • Case studies • Cohort studies • Case-control studies • Qualitative studies • Mixed methods studies • Diagnostic test accuracy studies	• Meta-reviews (reviews of reviews), systematic reviews, scoping reviews, umbrella reviews • Opinion pieces, editorials, and letters to the editor that do not contain empirical data • Abstracts without full-text availability • Non-peer-reviewed publications
Publication Language	English	Language other than English

With the assistance of a public health informationist (LR), we searched the following electronic databases: PubMed, Embase, Global Health, and the Cochrane Library. In addition, we searched the WHO Regional Libraries and Africa-Wide Information to ensure geographic representation from high TB burden regions. Finally, we searched PsycINFO given its comprehensive coverage of psychological and mental health research. The search was conducted on March 31, 2025 (search criteria found in S1 Text).

### Study selection and data charting

All search results were imported into Covidence, which we used for deduplication, screening, and data extraction. Two reviewers (combinations of AJG, PT, FS, and SI) reviewed titles and abstracts in parallel for inclusion. Reviewers were blinded to each other’s ratings except when resolving conflicts. All studies that passed initial screening had the full text reviewed by two reviewers (combinations of AJG, PT, FS, SI, AB, and DJ) for inclusion. Disagreements were resolved via discussion or by a third reviewer.

After an initial pilot of the data extraction tool within Covidence, we extracted pertinent study details including geography, setting (e.g., hospital, community, etc.), timeframe, study design, research aim, sample size, participant details (e.g., age, sex, HIV status, income, education, other mental health conditions), depression screening and diagnostic tool(s) used, intervention characteristics (if applicable), statistical methods used, outcomes assessed, and for qualitative studies themes identified including barriers and facilitators.

### Synthesis of results

We descriptively synthesized the data to map the range and frequency of methodological approaches used in the included studies. Results were organized using the RE-AIM framework (Reach, Effectiveness, Adoption, Implementation, Maintenance) to examine how research on depression among PWTB has been conducted and applied across contexts. No formal quality appraisal was performed. We explored reach in two ways. First, we evaluated the representativeness of included patient populations and the inclusion of high-risk groups (such as inclusion of persons living with HIV [PLHIV], drug-resistant TB, or pediatric cases). Second, we examined the extent to which studies explored both directions of the TB-depression relationship. We explored effectiveness by describing the range of study designs (e.g., cross-sectional, cohort studies, randomized trials). We also evaluated effectiveness as the reported effectiveness or efficacy of diagnostic measures and treatment interventions in terms of their ability to diagnose depression or improve TB- or depression-related outcomes. We defined adoption as the extent to which depression screening, diagnosis and treatment methodologies were adopted in different settings. For implementation, we evaluated barriers and facilitators to the implementation of depression-related care in TB treatment settings. We defined maintenance as the extent to which any depression-related methodologies for diagnosis or treatment were sustained or embedded within TB health care systems, including evaluation of long-term patient outcomes.

## Results

### Selection of sources

Our search identified 15,504 studies and after manual and automated duplicate removal, 9,985 unique records underwent title and abstract screening (Fig 1). From the screened records, we attempted to retrieve 576 full-text studies. Twenty-seven could not be retrieved, most of which were publications from before 1970 that were not accessible through available sources. Therefore, we assessed 549 full-text publications for inclusion, 184 of which met all eligibility criteria.

**Fig 1:**
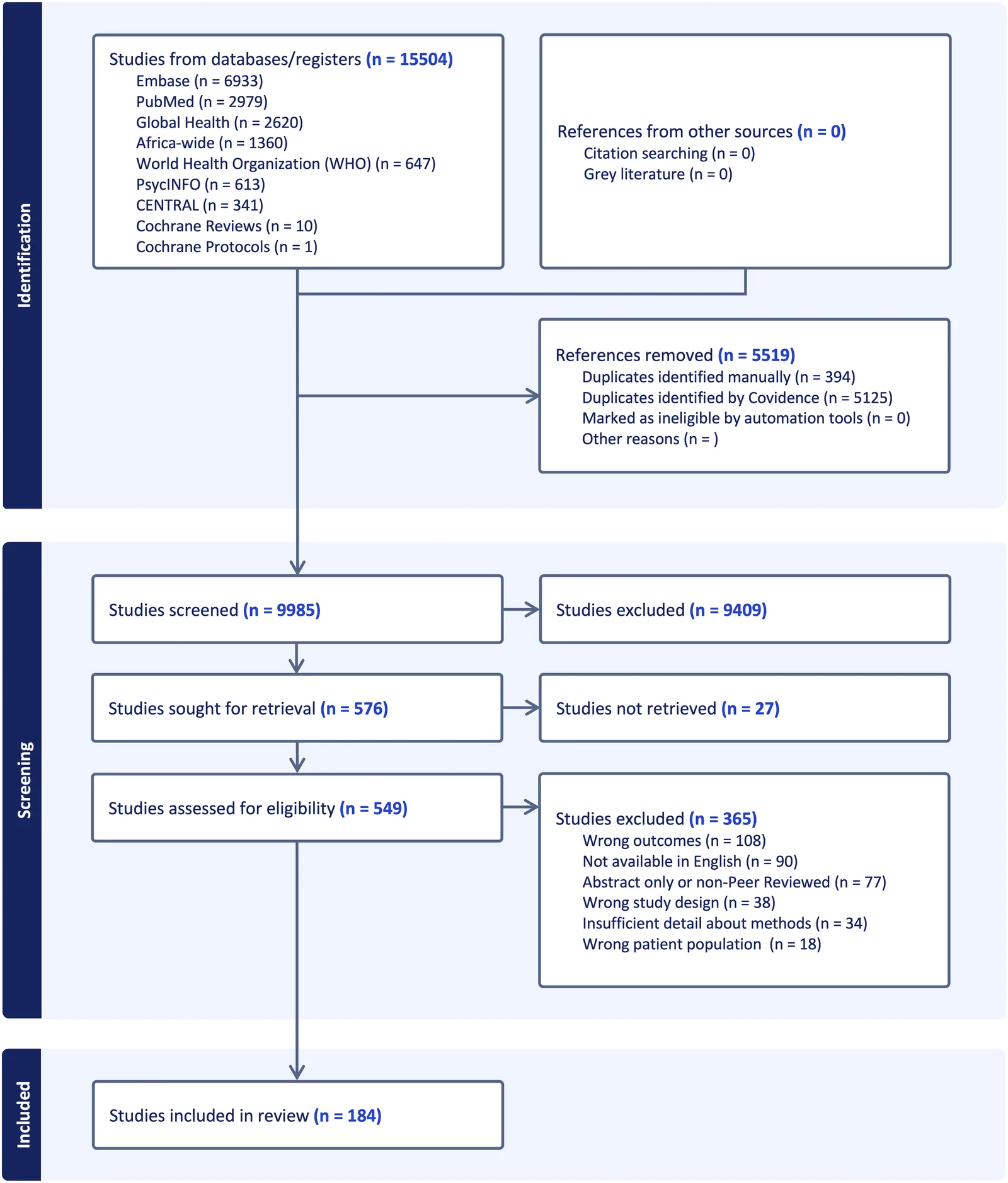
PRISMA flow diagram.

### Characteristics of sources of evidence

A complete description of the 184 included studies is available in S1 Table. Included studies were published between 1948 and 2025; 60% (n = 110) were published between 2020 and 2025. While studies were included from all regions of the world, more than a third of studies originated from India (n = 47, 26%) and China (n = 22, 12%) (Fig 2). More than half of the WHO-defined high-TB-burden countries (17 of 30) were represented by at least one unique study, with one large multi-site study involving 48 low- and middle-income countries.

**Fig 2:**
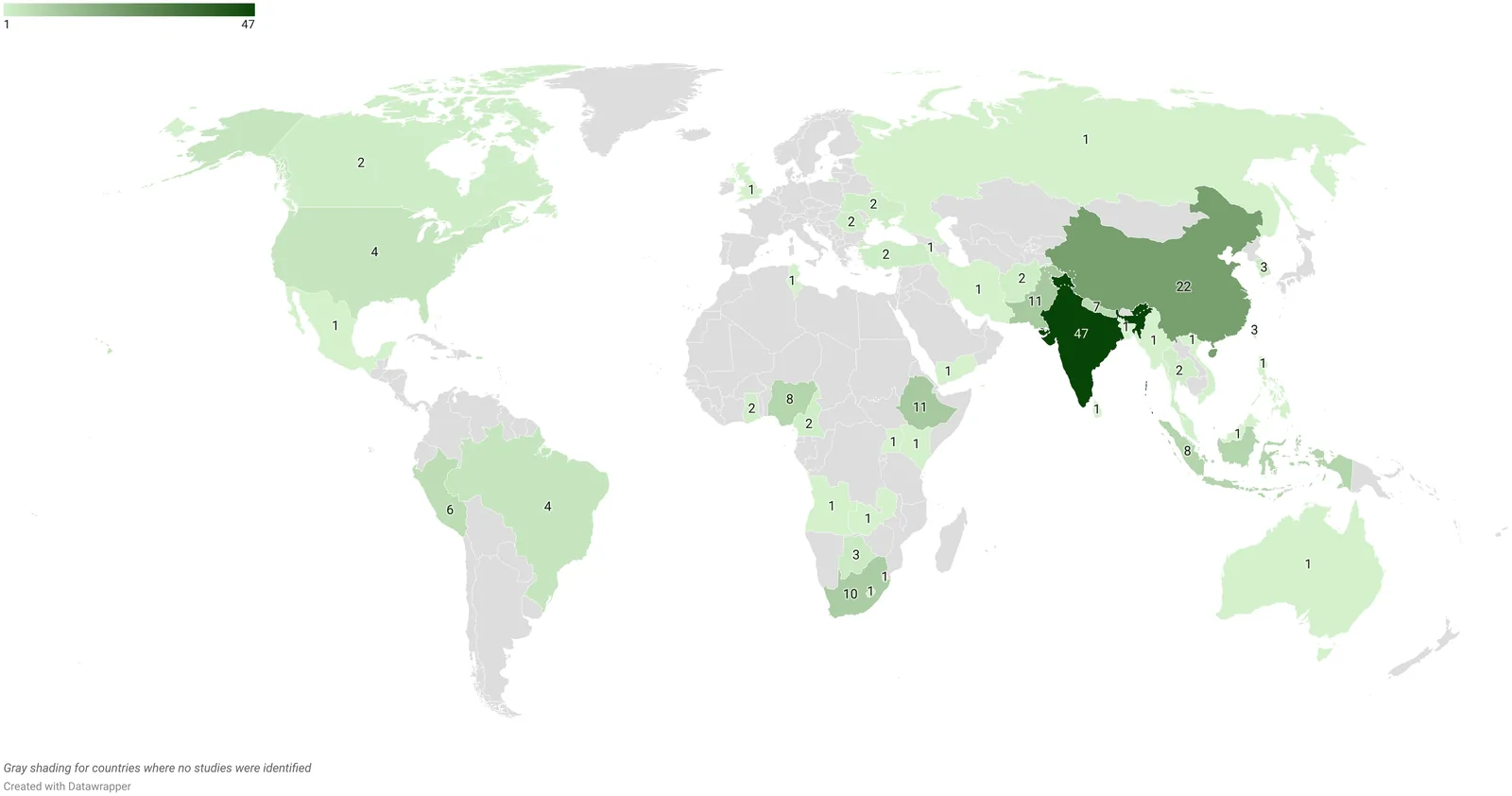
Geographic Distribution of Included Studies*. Legend: Heat map displaying the number of studies per country represented in the scoping review (n = 184). Countries with darker shading contributed a larger number of studies while gray shading indicates no studies from that country. *Map created using Datawrapper; base map data Natural Earth, free vector and raster map data.

### Reach

Most studies reported one or more TB types with 104 reporting on pulmonary TB (PTB), 52 on extrapulmonary (EP-TB) TB, and 57 on multidrug-resistant (MDR) or extensively drug-resistant (XDR) TB. Over one-third of studies (n = 63, 34%) enrolled patients representing multiple TB categories. Twenty-eight studies (15%) did not specify the type of TB included, reporting only that the study population consisted of “TB patients” without further detail. Fifty-eight studies (31%) included PLHIV. Few studies reported on pediatric populations (n = 24, 13%) with most including older adolescents (age ≥ 15), if children were included at all.

We identified only two studies [19,20] that evaluated the risk of TB among people experiencing depression. The first was a nationwide cohort study conducted in South Korea that demonstrated modestly higher incidence of TB among those experiencing depression compared to those who were not, though this association was not statistically significant (incidence rate ratio (IRR) 1.14, 95% CI 0.89-1.45) [19]. The second was a nationwide cohort study conducted in Taiwan that also demonstrated a higher incidence of PTB among those experiencing depression (IRR 1.16, 95% CI 1.12-1.21) [20]. All other studies examined the opposite direction of the relationship, assessing depression among PWTB, either its prevalence or the risk of developing depressive symptoms.

### Effectiveness

No study reported on diagnostic effectiveness of various tools or approaches for identifying depression; most studies used screening tools as opposed to diagnostic measures (Table 2). Only 28 studies (15%) used a diagnostic approach such as psychiatric interview, MINI (Mini-International Neuropsychiatric Interview), CIDI (Composite International Diagnostic Interview), ICD-10 (International Classification of Diseases, 10^th^ Revision) or DSM-IV (Diagnostic and Statistical Manual of Mental Disorders). Of the screening tools used, 40% (n = 72) utilized the PHQ-9 (Patient Health Questionnaire-9) with an additional 42% (n = 78) using the BDI (Beck’s Depression Inventory, n = 19), CES-D (Center for Epidemiologic Studies Depression Scale, n = 18), HADS (Hospital Anxiety and Depression Scale, n = 18), HAM-D (Hamilton Depression Rating Scale, n = 17), or SDS (Zung Self-Rating Depression Scale, n = 10) screening tools. Seven studies (4%) used more than one screening tool to classify depression including both the PHQ-9 and PHQ-2 (n = 2), PHQ-9 and HADS (n = 1), PHQ-9 and BDI (n = 1), PHQ-9 and Hopkins Symptom Checklist (n = 1), PHQ-2 and CES-D (n = 1), and BDI and HAM-D (n = 1). One study used the Children’s Depression Inventory scale as their entire study population was < 18 years old [21]. Other studies that included children did not report using screening or diagnostic tools specific for those under the age 18.

**Table 2:** Depression tools utilized.

Depression Tool Utilized	Type of Tool	Number of Studies n (%)^#^
PHQ-9	Screening	72 (39)
BDI	Screening	19 (10)
CES-D	Screening	18 (10)
HADS	Screening	18 (10)
HAM-D	Screening	13 (7)
MINI or MINI-Plus	Diagnostic	13 (7)
SDS	Screening	10 (5)
ICD-10 Criteria	Diagnostic	8 (4)
Psychiatrist Evaluation	Diagnostic	6 (3)
PHQ-2	Screening	3 (2)
DSM-IV or DSM-V	Diagnostic	3 (2)
CIDI	Diagnostic	2 (1)
K-10	Screening	2 (1)
ICD-9 Criteria	Diagnostic	2 (1)
HSCL	Screening	2 (1)
PHQ-4	Screening	1 (0.5)
MMPI	Screening	1 (0.5)
GHQ	Screening	1 (0.5)
SF12v2	Screening	1 (0.5)
Present State Examination	Screening	1 (0.5)
SCL	Screening	1 (0.5)
CDI	Screening	1 (0.5)

**Legend:** ^#^Studies may have used ≥1 tool. Therefore, percentage totals do not sum to 100; **Abbreviations:** PHQ-9, Patient Health Questionnaire-9; BDI, Beck’s Depression Inventory; CES-D, Center for Epidemiologic Studies Depression Scale; HADS, Hospital Anxiety and Depression Scale; HAM-D, Hamilton Depression Rating Scale; MINI, Mini International Neuropsychiatric Interview; SDS, Zung Self-Rating Depression Scale; ICD-10, International Classification of Diseases, 10^th^ Revision; PHQ-2, Patient Health Questionnaire-2; DSM, Diagnostic and Statistical Manual of Mental Disorders; CIDI, Composite International Diagnostic Interview; K-10, Kessler Psychological Distress Scale; ICD-9, International Classification of Diseases, 9^th^ Revision; HSCL, Hopkins Symptom Checklist; PHQ-4, Patient Health Questionnaire-4; MMPI, Minnesota Multiphasic Personality Inventory; GHQ, General Health Questionnaire; SF12v2, Short-Form 12-Item Survey version 2; SCL, Syndrome Check-List; CDI, Children’s Depression Inventory;

As seen in Fig 3, most studies identified were cross sectional (n = 121, 65%). Thirty-one studies (17%) were cohort studies, and randomized controlled trials (RCTs) represented only 4% of identified studies (n = 8). The final 13% (n = 24) of studies were case reports or series, qualitative, case control, quasi-experimental, single arm interventional studies, or mixed-methods studies.

**Fig 3:**
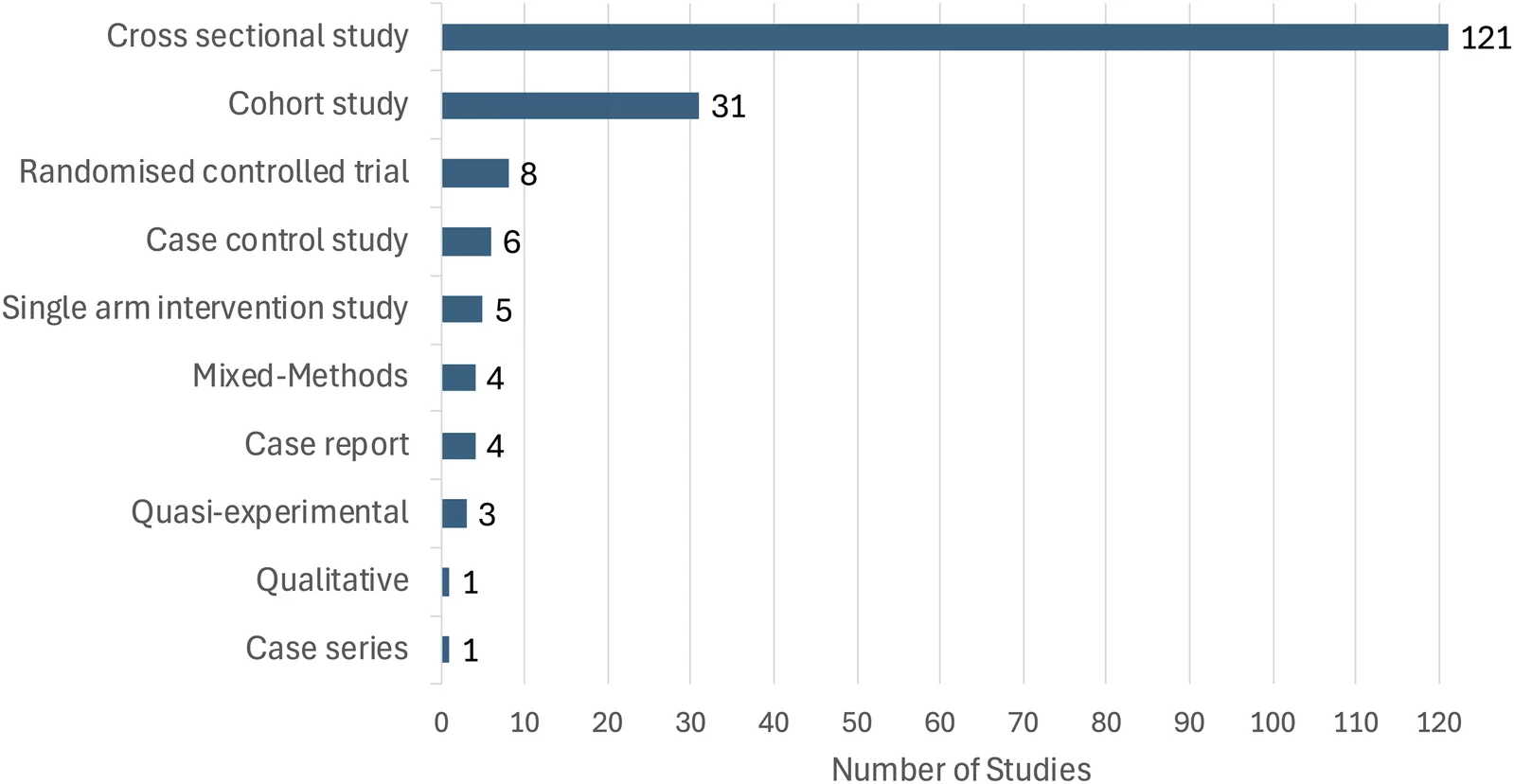
Study designs represented by included studies.

Of the 8 RCTs [22–29], six trials took place in China, one in Pakistan, and one in Armenia. Only one reported including PLHIV and none reported the inclusion of children. Seven compared psychological interventions to standard of care, while one study evaluated a pharmacological intervention [23], finding that vitamin D supplementation did not significantly impact mean scores on the BDI. Among the seven psychological interventions tested, all but two showed statistically significant changes in the outcomes of interest. Of the two that did not find a statistically significant result, one evaluated a family supported treatment model supplemented by a single education and psychological counseling session amongst those with MDR-TB (p = 0.10) [25]. The other was similarly aimed at patients with MDR-TB and consisted of a pharmacist led health education and counseling intervention supplemented by a monthly food basket and transport fees [22]. This study did not test for statistical significance but findings after 10 months of support showed fewer participants in the moderate or severe depression categories (as defined by BDI) compared to those in the control group (4 out of 35 participants vs 35 out of 35 participants).

The remaining five RCTs demonstrated effectiveness based on their primary outcomes. One demonstrated that among elderly TB patients (>65 years), a combination of psychotherapy, home visits, peer support, and psycho-educational workshops created a significantly faster decline in mean SDS scores over the course of six months compared to routine health education alone [24]. The second trial similarly demonstrated significant declines amongst in SDS score among inpatients with XDR-TB over time (p < 0.001) but also significantly higher treatment compliance (p < 0.05) in those receiving a targeted nursing intervention combined with psychological counseling [26]. The third (and largest) RCT, which had a total of 454 participants with PTB randomized to either usual care or cognitive behavioral therapy (CBT) delivered by general practitioners in community settings, demonstrated a significant improvement in PHQ-9 scores at two months (p < 0.001). In the fourth RCT, an early rehabilitation nursing intervention based on the Health Belief Model, initiated immediately after PTB diagnosis, was more effective than usual care [28]. Finally, a small RCT (n = 60) testing an integrated nursing and psychological intervention among those with both PTB and lung cancer demonstrated a significant difference in pre and post SDS scores (p < 0.001) [29].

### Adoption, implementation, and maintenance

Most studies took place in facilities such as hospitals, primary care clinics, or TB treatment centers, with far fewer conducted in community environments (n = 12). As stated above, almost all studies utilized screening tools rather than diagnostic tools for depression. These tools were often adopted for their feasibility and ease of administration in busy clinical environments, which may explain their widespread uptake across diverse geographic and resource settings. Overall, there was low adoption of depression related diagnostics and treatments.

Of the four studies that discussed implementation facilitators and barriers [30–33], three discussed how staffing shortages and an overburdened workforce with little time for additional tasks made the integration of depression screening and care more difficult [30,31,33]. Conversely, task-shifting depression screening to counselors or community health workers improved the feasibility of its implementation [33]. Additionally, two of the studies [30,31] discussed how mistrust of healthcare providers and fears about providing answers to mental health questions represented barriers to the acceptability of and ultimately implementation of depression screening into routine TB care.

No studies in this review reported evidence of maintenance of depression-related diagnosis or treatment methodologies within TB programs. Across the included studies, none described the continuation of screening or treatment activities beyond the research period, nor did any evaluate whether these approaches were integrated into routine TB care following study completion. Similarly, no study assessed long-term patient outcomes related to depression, including any outcomes after the completion of TB treatment.

## Discussion

This scoping review of 184 studies demonstrates that research on depression among PWTB has been dominated by cross-sectional prevalence studies, with relatively few employing longitudinal or interventional designs to understand the bidirectional nature of the TB-depression relationship. While screening tools such as the PHQ-9, HADS, and CES-D were widely adopted, the implementation of diagnostic interviews and depression treatment was limited, with only a few RCTs demonstrating modest benefits and none embedding care into routine TB services. Finally, no studies reported maintenance, as none assessed long-term sustainability, integration of depression care into TB programs, or patient outcomes beyond TB treatment completion, highlighting a major gap in our understanding of durable, system-level responses to the TB-depression syndemic.

Findings across the RE-AIM domains highlight opportunities to expand the reach of current research efforts. Many studies enrolled broad TB populations, including those with MDR, XDR, or those living with HIV, but other key populations that may be at high risk for depression, like pediatric populations, were not included in most studies. Moreover, few studies explored the reverse direction of the relationship (i.e., whether depression increases the risk of developing TB). Since most studies were cross-sectional, there remains a need for longitudinal designs that can more fully capture patient trajectories, including journeys after the completion of TB treatment, and more fully define the bidirectional nature of TB and depression. Additionally, a worryingly low proportion of studies utilized gold-standard diagnostic methods such as psychiatric evaluations or DSM or ICD-based assessments, and the overwhelming reliance on screening tools limits our ability to determine the true burden of depression among PWTB. This limitation is particularly important because most depression screening instruments were originally developed and validated in general populations rather than in individuals with active TB. The substantial overlap between the somatic manifestations of TB and symptoms commonly assessed by depression screening tools, including fatigue, appetite loss, weight loss, and sleep disturbance, raises the possibility of symptom misclassification during active TB illness. Few studies reported validation of these instruments specifically among PWTB or considered whether the timing of depression assessment relative to TB treatment initiation might influence the interpretation of screening results. Consequently, estimates of depression prevalence among PWTB should be interpreted cautiously until more rigorous validation studies are conducted in this population if screening tools are to be used as the primary resource for depression prevalence measurement. Beyond limiting estimates of true prevalence, the reliance on screening tools without confirmatory diagnostic assessment raises concern about the potential for under- or over-diagnosis of depression among PWTB at scale. On one hand, overlapping somatic symptoms of TB may increase depression screening scores independent of depressive illness, resulting in the overestimation of the prevalence of clinically significant depression. Conversely, if depression manifests differently among PWTB or if standard screening thresholds do not perform similarly in this population, existing instruments may fail to identify clinically important depression or under classify the severity of depressive symptoms, resulting in missed cases. While screening instruments are valuable for identifying individuals at risk, they are not designed to establish clinical diagnoses and may fail to capture more complex or context-specific presentations of depression or lack the specificity necessary for a depression diagnosis. As the included studies did not evaluate the diagnostic accuracy or optimal thresholds of depression screening instruments among PWTB, the magnitude and direction of this potential misclassification remain unknown and represents a critical cap for future research. Furthermore, these findings highlight the importance of pairing screening approaches with appropriate diagnostic pathways to ensure that identification of depressive symptoms translates into meaningful clinical care. Finally, the over-representation of studies conducted in China and India suggests geographic clustering in research (and our corresponding knowledge), with fewer contributions from many high-burden countries in sub-Saharan Africa, Central Asia, and Eastern Europe. Taken together, these findings emphasize that expanding both population and geographic coverage, alongside the use of more rigorous and diverse methodological approaches, will be essential for strengthening the evidence base.

The overall evidence base for effective interventions to mitigate depression among people with TB remains thin. Among the few RCTs identified, most evaluated psychological interventions, with several demonstrating reductions in depressive symptoms or improvements in TB treatment adherence. However, these trials were small, geographically concentrated, and generally excluded complex TB populations, such as those with MDR, XDR, or multiple co-morbidities such as diabetes or HIV, limiting generalizability. Pharmacologic and nutritional interventions were nearly absent from this literature, and no studies considered potential drug-drug interactions or the integration of common depression treatments with TB regimens.

Adoption, implementation, and maintenance domains were similarly constrained. While screening was common, the integration of diagnostic and treatment approaches for depression into TB care was rare, and interventions were often delivered by research teams rather than embedded within existing health systems. Implementation barriers included limited staffing, insufficient training, stigma, and logistical constraints that reduced the feasibility of sustained depression care but given the lack of evaluation of long-term outcomes, the true feasibility of integrated depression and TB care remains unevaluated. A small number of studies identified facilitators that may provide preliminary insights into how depression care could be integrated into TB programs. These included task-shifting to community health workers, embedding mental health assessments into routine workflows, and leveraging family or peer support. However, these facilitators similarly remain unevaluated in experimental studies, and implementation outcomes beyond acceptability and feasibility – such as fidelity, sustainability, and cost – were not measured in any of our identified studies. Regarding maintenance, none of the included studies assessed whether depression screening or treatment interventions persisted beyond TB treatment completion or if they generated long-term improvements in patient outcomes (such as decreases in recurrent TB, improvements in health-related quality of life, etc.). This lack of data on sustainability limits understanding of how mental health care might realistically be integrated into TB care systems, particularly in resource-constrained settings. Without evidence on how interventions can be maintained, scaled, or institutionalized, the field remains ill-equipped to support the development of durable, system-level approaches to addressing comorbid depression among PWTB.

Several limitations to this scoping review should be noted. First, the review was restricted to English-language publications, which may have excluded relevant studies from non-English speaking high-burden countries. A total of 90 non-English records progressed through title and abstract screening but were excluded at full-text screening due to this restriction. While we are unable to determine how many of these studies would have ultimately met all inclusion criteria, their exclusion may have limited conclusions about the geographic representation of the review. Anecdotally, many of these records were Spanish-, Portuguese-, and Russian-language publications, suggesting a potential misclassification of the representation of Latin America and Eastern Europe. Despite this limitation, there was at least one included study from each continent with just over half of the 30 high TB burden countries represented leading us to believe that the primary conclusions regarding methodological patterns are unlikely to be substantially affected. Nonetheless, the findings should be interpreted with caution in terms of geographic completeness. Second, this review focused exclusively on the peer-reviewed literature and excluded gray literature, which may have limited out ability to fully assess adoption and maintenance. These domains are often better documented in non-peer-reviewed sources such as TB program reports, implementation guidelines, and other programmatic materials. Third, the review relied on information as reported in published articles, which varied considerably in depth and clarity. Inconsistent reporting of methodological details, such as how screening tools were administered, how interventions were delivered, or even how statistical analyses were conducted, may have led to an underestimation or misclassification of methodological practices or effectiveness across studies.

This scoping review has also had several strengths. It followed a rigorous, published protocol [17], adhered to PRISMA-ScR guidelines, and incorporated a comprehensive search strategy across multiple databases without date restrictions. Additionally, the use of the RE-AIM framework provided a structured and systematic approach for evaluating methodological gaps and identifying opportunities for future research.

In summary, this scoping review highlights the need for methodologically rigorous studies that move beyond prevalence estimates to explore causal pathways, temporal relationships, and the bidirectionality of the TB-depression syndemic. Additional longitudinal cohort studies and mixed-methods designs could provide nuanced insights into how symptoms evolve over time, while RCTs, particularly those conducted in diverse geographic settings, could evaluate the effectiveness of pharmacological and psychological treatments adapted for TB care. Routine screening for depression is feasible given the widespread use in research contexts; however, screening must be paired with referral pathways for appropriate diagnosis and treatment to avoid perpetuating unmet mental health needs. Task-shifting strategies and brief, scalable psychological interventions may offer practical routes for expanding access to depression care for PWTB, but further research is needed to evaluate how to best integrate care for these comorbid conditions. By confronting these knowledge gaps directly, future research could advance more equitable, comprehensive, and sustainable care for people affected by TB and depression.

## Supporting information

S1 Text Search criteria by database. (DOCX)

S1 Data Complete data extraction. (XLSX)

S1 Checklist PRISMA-ScR checklist. (DOCX)

S1 Table Overview of included studies. (DOCX)

## References

[1] World Health Organization. Global tuberculosis report 2025. Geneva, Switzerland: World Health Organization; 2025. https://www.who.int/teams/global-programme-on-tuberculosis-and-lung-health/tb-reports/global-tuberculosis-report-2025

[2] Kim J, Keshavjee S, Atun R. Health systems performance in managing tuberculosis: analysis of tuberculosis care cascades among high-burden and non-high-burden countries. J Glob Health. 2019;9(1):010423. doi: 10.7189/jogh.09.010423 31263546 PMC6592589

[3] Armstrong-Hough M, Turimumahoro P, Meyer AJ, Ochom E, Babirye D, Ayakaka I, et al. Drop-out from the tuberculosis contact investigation cascade in a routine public health setting in urban Uganda: A prospective, multi-center study. PLoS One. 2017;12(11):e0187145. doi: 10.1371/journal.pone.0187145 29108007 PMC5673209

[4] Jarde A, Romano E, Afaq S, Elsony A, Lin Y, Huque R, et al. Prevalence and risks of tuberculosis multimorbidity in low-income and middle-income countries: a meta-review. BMJ Open. 2022;12(9):e060906. doi: 10.1136/bmjopen-2022-060906 36175100 PMC9528681

[5] Samuels JP, Sood A, Campbell JR, Ahmad Khan F, Johnston JC. Comorbidities and treatment outcomes in multidrug resistant tuberculosis: A systematic review and meta-analysis. Sci Rep. 2018;8(1):4980. doi: 10.1038/s41598-018-23373-3 PMC5862834 29563561

[6] Khattak M, Rehman AU, Muqaddas T, Hussain R, Rasool MF, Saleem Z, et al. Tuberculosis (TB) treatment challenges in TB-diabetes comorbid patients: A systematic review and meta-analysis. Ann Med. 2024;56(1):2313683. doi: 10.1080/07853890.2024.2313683 38346381 PMC10863515

[7] Nowiński A, Wesołowski S, Korzeniewska-Koseła M. The impact of comorbidities on tuberculosis treatment outcomes in Poland: A national cohort study. Front Public Health. 2023;11:1253615. doi: 10.3389/fpubh.2023.1253615 37732096 PMC10508909

[8] Simon GE, Moise N, Mohr DC. Management of depression in adults: A review. JAMA. 2024;332(2):141–52. 10.1001/jama.2024.5756 38856993

[9] Duko B, Bedaso A, Ayano G. The prevalence of depression among patients with tuberculosis: A systematic review and meta-analysis. Ann Gen Psychiatry. 2020;19:30. doi: 10.1186/s12991-020-00281-8 32419837 PMC7206806

[10] Alemu WG, Zeleke TA. Prevalence of depression in people with tuberculosis in East Africa: A systematic review and meta-analysis. Afr Health Sci. 2023;23(1):231–40. doi: 10.4314/ahs.v23i1.25 37545972 PMC10398488

[11] Samal J, Dehury RK, Thomas MB, Singh H. Prevalence and predictors of depression in tuberculosis patients in india: A systematic review and meta-analysis. Discov Ment Health. 2025;5(1):104. doi: 10.1007/s44192-025-00248-9 40650839 PMC12255643

[12] Ruiz-Grosso P, Cachay R, de la Flor A, Schwalb A, Ugarte-Gil C. Association between tuberculosis and depression on negative outcomes of tuberculosis treatment: A systematic review and meta-analysis. PLoS One. 2020;15(1):e0227472. doi: 10.1371/journal.pone.0227472 31923280 PMC6953784

[13] Molebatsi K, Wang Q, Dima M, Ho-Foster A, Modongo C, Zetola N, et al. Depression and delayed tuberculosis treatment initiation among newly diagnosed patients in Botswana. Glob Public Health. 2021;16(7):1088–98. doi: 10.1080/17441692.2020.1826049 32991275 PMC8005506

[14] Kumpuangdee S, Roomruangwong C, Sophonphan J, Ohata PJ, Suwanpimolkul G. Prevalence of depression and anxiety in pulmonary tuberculosis patients and its association with unsuccessful treatment outcome: A prospective cohort study. Indian J Tuberc. 2023;70(3):297–302. doi: 10.1016/j.ijtb.2022.05.007 37562903

[15] Lara-Espinosa JV, Hernández-Pando R. Psychiatric problems in pulmonary tuberculosis: Depression and anxiety. J Tuberc Res. 2021;09(01):31–50.

[16] Levac D, Colquhoun H, O’Brien KK. Scoping studies: Advancing the methodology. Implement Sci. 2010;5:69. doi: 10.1186/1748-5908-5-69 20854677 PMC2954944

[17] Gupta AJ, Turimumahoro P, Rosman L, Golub JE, Dowdy DW. Methodologies for studying depression in persons living with tuberculosis: Protocol for a scoping review. PLoS One. 2025;20(7):e0322753. doi: 10.1371/journal.pone.0322753 40690459 PMC12279151

[18] Tricco AC, Lillie E, Zarin W, O’Brien KK, Colquhoun H, Levac D, et al. PRISMA Extension for Scoping Reviews (PRISMA-ScR): Checklist and explanation. Ann Intern Med. 2018;169(7):467–73. doi: 10.7326/M18-0850 30178033

[19] Oh KH, Choi H, Kim EJ, Kim HJ, Cho SI. Depression and risk of tuberculosis: a nationwide population-based cohort study. Int J Tuberc Lung Dis. 2017;21(7):804–9. doi: 10.5588/ijtld.17.0038 28633706

[20] Cheng K-C, Liao K-F, Lin C-L, Lai S-W. Increased risk of pulmonary tuberculosis in patients with depression: A cohort study in Taiwan. Front Psychiatry. 2017;8:235. doi: 10.3389/fpsyt.2017.00235 29180971 PMC5694036

[21] Mihailov OM, Ciubară A, Luțenco V, Țocu G, Stavăr Matei L, Mihailov R. Clinical strategies for identifying pediatric patients with tuberculosis at risk of developing depressive disorders. Clin Pract. 2024;14(6):2385–409. doi: 10.3390/clinpract14060187 39585015 PMC11586971

[22] Kaukab I, Nasir B, Abrar M, Muneer S, Kanwal N, Shah S. Effect of pharmacist-led patient education on management of depression in drug resistance tuberculosis patients using cycloserine. A Propective Study. 2015;34(7):1403–10.

[23] Zhang L, Wang S, Zhu Y, Yang T. Vitamin D3 as adjunctive therapy in the treatment of depression in tuberculosis patients: a short-term pilot randomized double-blind controlled study. Neuropsychiatr Dis Treat. 2018;14:3103–9. doi: 10.2147/NDT.S183039 30532541 PMC6241718

[24] Li X, Wang B, Xu Y, Chen Y, Zhang Y, Huang J, et al. Comprehensive intervention for anxiety and depression among the community elderly with tuberculosis. Basic and Appl Soc Psychol. 2019;41(3):179–87. doi: 10.1080/01973533.2019.1600519

[25] Khachadourian V, Truzyan N, Harutyunyan A, Petrosyan V, Davtyan H, Davtyan K, et al. People-centred care versus clinic-based DOT for continuation phase TB treatment in Armenia: A cluster randomized trial. BMC Pulm Med. 2020;20(1):105. doi: 10.1186/s12890-020-1141-y 32334553 PMC7183136

[26] Lu Y, Wang H, Zhu J, Wang N, Cui D, Li L. Effect of targeted nursing intervention plus psychological counseling on quality of life, negative emotions, and complications in patients with extensively drug-resistant tuberculosis. Am J Transl Res. 2021;13(12):13950–8. 35035736 PMC8748094

[27] Zuo X, Dong Z, Zhang P, Zhang P, Zhu X, Qiao C, et al. Cognitive-behavioral therapy on psychological stress and quality of life in subjects with pulmonary tuberculosis: a community-based cluster randomized controlled trial. BMC Public Health. 2022;22(1):2160. doi: 10.1186/s12889-022-14631-6 36419020 PMC9686076

[28] Xie L, Wang D, Xie X. Development and evaluation of an early rehabilitation nursing program for patients with pulmonary tuberculosis. Medicine (Baltimore). 2023;102(36):e34991. doi: 10.1097/MD.0000000000034991 37682157 PMC10489242

[29] Yuan W, Chai L, Wang J, Liu C, Jiang Z, Deng M, et al. Integrated nursing and psychological intervention for tuberculosis complicated by lung cancer: clinical efficacy. Altern Ther Health Med. 2024;30(12):274–9. 38518147

[30] Afaq S, Ayub A, Faisal MR, Nisar Z, Zala, Rehman AU, et al. Depression care integration in tuberculosis services: A feasibility assessment in Pakistan. Health Expect. 2024;27(1):e13985. doi: 10.1111/hex.13985 39102704 PMC10849063

[31] Apang O, Shringarpure K, Laxmeshwar C, Nuken A, Yubey M, Nyori M. Screening and management of depression among patients on TB treatment in Papumpare district, Arunachal Pradesh, India. Indian J Tuberc. 2024;71(S2):S155-61. 10.1016/j.ijtb.2024.05.009 39370176

[32] Stavrou P, Sgouros S. Frontal lobe tuberculoma masquerading as postnatal depression. Br J Neurosurg. 2002;16(1):74. doi: 10.1080/02688690120114278 11926473

[33] Walker IF, Khanal S, Hicks JP, Lamichhane B, Thapa A, Elsey H, et al. Implementation of a psychosocial support package for people receiving treatment for multidrug-resistant tuberculosis in Nepal: A feasibility and acceptability study. PLoS One. 2018;13(7):e0201163. doi: 10.1371/journal.pone.0201163 30048495 PMC6062069

