# Supplementary material for: Methodologies for studying depression in persons with tuberculosis: A scoping review
Source: PLOS Glob Public Health. 2026 Sep 28;6(9):e0007388. doi: 10.1371/journal.pgph.0007388 (PMC13618889; doi:10.1371/journal.pgph.0007388)
Supplement: Supplementary file 1 [file pgph.0007388.s001.docx]

**S1 Text:** Search Criteria by Database

**PubMed**

| #1 | ("Tuberculosis"[MeSH] OR "Mycobacterium Tuberculosis"[MeSH] OR tuberculosis[tiab] OR TB[tiab] OR XMTB[tiab]) |
| --- | --- |
| #2 | "Depression"[MeSH] OR "Depressive Disorder"[MeSH] OR "Mental Health"[MeSH] OR "Psychological Well-Being"[Mesh] OR "Mood Disorders"[Mesh:NoExp] OR "Psychological Distress"[Mesh] OR "Psychosocial Intervention"[Mesh] OR "Affective Disorders, Psychotic"[Mesh] OR "Mental Disorders"[Mesh:NoExp] OR depression[tiab] OR depressive[tiab] OR depressed[tiab] OR "mental health"[tiab] OR "emotional health"[tiab] OR "mental illness*"[tiab] OR "well being"[tiab] OR "wellbeing"[tiab] OR "mood disorder*"[tiab] OR "psychological distress*"[tiab] OR "psychosocial"[tiab] OR "affective disorder*"[tiab] OR "mental disorder*"[tiab] OR "psychiatric symptom*"[tiab] OR "psychiatric disorder*"[tiab] |
|  | #1 AND #2 |

**Cochrane Library**

#1	MeSH descriptor: [Tuberculosis] explode all trees

#2	(tuberculos* OR TB OR XMTB):ti,ab,kw

#3	#1 OR #2

#4	MeSH descriptor: [Depression] explode all trees

#5	MeSH descriptor: [Depressive Disorder] explode all trees

#6	MeSH descriptor: [Mental Health] explode all trees

#7	MeSH descriptor: [Psychological Well-Being] explode all trees

#8	MeSH descriptor: [Mood Disorders] this term only

#9	MeSH descriptor: [Psychological Distress] explode all trees

#10	MeSH descriptor: [Psychosocial Intervention] explode all trees

#11	MeSH descriptor: [Affective Disorders, Psychotic] explode all trees

#12	MeSH descriptor: [Mental Disorders] this term only

#13	(depression OR depressive OR depressed OR "mental health" OR "emotional health" OR (mental NEXT/1 illness*) OR "well being" OR "wellbeing" OR (mood NEXT/1 disorder*) OR (psychological NEXT/1 distress*) OR "psychosocial" OR (affective NEXT/1 disorder*) OR (mental NEXT/1 disorder*) OR (psychiatric NEXT/1 symptom*) OR (psychiatric NEXT/1 disorder*)):ti,ab,kw

#14	{OR #4-#13}

#15	#3 AND #14

**Embase.com**

1.	('tuberculosis'/exp) OR (tuberculos* OR TB OR XMTB):ti,ab,kw

2.	('depression'/exp OR 'mental health'/exp OR 'mood disorder'/de OR 'distress syndrome'/exp OR 'psychosocial intervention'/exp OR 'major affective disorder'/exp OR 'minor affective disorder'/exp) OR (depression OR depressive OR depressed OR "mental health" OR "emotional health" OR "mental illness*" OR "well being" OR "wellbeing" OR "mood disorder*" OR "psychological distress*" OR "psychosocial" OR "affective disorder*" OR "mental disorder*" OR "psychiatric symptom*" OR "psychiatric disorder*"):ab,ti,kw

3.	#1 AND #3

**GlobalHealth**

| 1 | exp tuberculosis/ or (tuberculos* OR TB OR XMTB).ti,ab. |
| --- | --- |
| 2 | (exp depression/ or exp mental health/ or mental disorders/) or (depression or epressive or depressed or "mental health" or "emotional health" or "mental illness*" or "well being" or "wellbeing" or "mood disorder*" or "psychological distress*" or "psychosocial" or "affective disorder*" or "mental disorder*" or "psychiatric symptom*" or "psychiatric disorder*").ti,ab. |
| 3 | 1 and 2 |

**PsycInfo**

| S1 | DE "Tuberculosis" OR DE "Pulmonary Tuberculosis" |
| --- | --- |
| S2 | **TI** (tuberculos* OR TB OR XMTB) OR **AB** (tuberculos* OR TB OR XMTB) |
| S3 | S1 OR S2 |
| S4 | (DE "Depression (Emotion)") OR (DE "Major Depression" OR DE "Anaclitic Depression" OR DE "Dysthymic Disorder" OR DE "Endogenous Depression" OR DE "Reactive Depression" OR DE "Recurrent Depression" OR DE "Treatment Resistant Depression") OR DE "Mental Health" OR DE "Emotional Health" OR (DE "Affective Disorders" OR DE "Disruptive Mood Dysregulation Disorder" OR DE "Persistent Depressive Disorder") OR (DE "Psychosocial Interventions" OR DE "Cognitive Stimulation Therapy") |
| S5 | **TI** (depression OR depressive OR depressed OR "mental health" OR "emotional health" OR "mental illness*" OR "well being" OR "wellbeing" OR "mood disorder*" OR "psychological distress*" OR "psychosocial" OR "affective disorder*" OR "mental disorder*" OR "psychiatric symptom*" OR "psychiatric disorder*") OR **AB** (depression OR depressive OR depressed OR "mental health" OR "emotional health" OR "mental illness*" OR "well being" OR "wellbeing" OR "mood disorder*" OR "psychological distress*" OR "psychosocial" OR "affective disorder*" OR "mental disorder*" OR "psychiatric symptom*" OR "psychiatric disorder*") |
| S6 | S4 OR S5 |
| S7 | S3 AND S6 |

**WHO Regional Library Search- Global Index Medicus**

- LILACS
- WPRIM
- IMSEAR
- IMEMR
- AIM

(MH:C01.150.252.410.040.552.846$ OR tuberculos$ OR TB OR XMTB) **AND** (MH:F01.145.126.350$ OR MH:F01.470.282$ OR MH:F03.600.300$ OR MH:F02.418$ OR MH:N01.400.500$ OR MH:SP2.770.825$ OR MH:F01.145.677.500$ OR MH:I01.800.500$ OR MH:K01.752.400.750.500$ OR MH:N06.850.505.400.425.837.500$ OR MH: F03.600 OR MH:F01.470.315$ OR MH:F04.754.715$ OR MH:F03.700.150$ OR MH:F03 OR depression OR depressive OR depressed OR "mental health" OR "emotional health" OR (mental illness$) OR "well being" OR "wellbeing" OR (mood disorder$) OR "psychological distress" OR "psychosocial" OR (affective disorder$) OR (mental disorder$) OR (psychiatric symptom$) OR (psychiatric disorder$))

**Africa-Wide Information**

| S1 | **TI** (tuberculos* OR TB OR XMTB) OR **AB** (tuberculos* OR TB OR XMTB) |
| --- | --- |
| S2 | **TI** (depression OR depressive OR depressed OR "mental health" OR "emotional health" OR "mental illness*" OR "well being" OR "wellbeing" OR "mood disorder*" OR "psychological distress*" OR "psychosocial" OR "affective disorder*" OR "mental disorder*" OR "psychiatric symptom*" OR "psychiatric disorder*") OR **AB** (depression OR depressive OR depressed OR "mental health" OR "emotional health" OR "mental illness*" OR "well being" OR "wellbeing" OR "mood disorder*" OR "psychological distress*" OR "psychosocial" OR "affective disorder*" OR "mental disorder*" OR "psychiatric symptom*" OR "psychiatric disorder*") |
| S3 | S1 AND s2 |
