# Supplementary material for: Methodologies for studying depression in persons with tuberculosis: A scoping review
Source: PLOS Glob Public Health. 2026 Sep 28;6(9):e0007388. doi: 10.1371/journal.pgph.0007388 (PMC13618889; doi:10.1371/journal.pgph.0007388)
Supplement: Supplementary file 3 [file pgph.0007388.s004.docx]

**S1 Table.** Overview of Included Studies

| **First Author** | **Year of Publication** | **Journal Name** | **Title** | **Study design** | **Country** |
| --- | --- | --- | --- | --- | --- |
| Abdurahman, S | 2022 | Neuropsychiatric Disease and Treatment | Magnitude of Depression and Associated Factors Among Patients on Tuberculosis Treatment at Public Health Facilities in Harari Regional State, Eastern Ethiopia: Multi-Center Cross-Sectional Study | Cross sectional study | Ethiopia |
| Adejumo, Olusola Adedeji | 2024 | Tropical Medicine & International Health | Association between experienced stigma, anxiety, depression and loneliness among people with drug-resistant tuberculosis in Lagos Nigeria: The moderating role of social support | Cross sectional study | Nigeria |
| Afaq, Saima | 2023 | Health Expectations | Depression care integration in tuberculosis services: A feasibility assessment in Pakistan | Mixed-Methods | Pakistan |
| Ageel, Rushdhan Husnee | 2024 | Industrial Psychiatry Journal | Tuberculosis-induced mood disorder | Case report | India |
| Aghanwa, HS | 1998 | Journal of Psychosomatic Research | Demographic/Socioeconomic Factors in Mental Disorders Associated with Tuberculosis in Southwest Nigeria | Cohort study | Nigeria |
| Albee, George W | 1948 | American Review of Tuberculosis | Psychological concomitants of pulmonary tuberculosis | Case control study | United States |
| Alinaitwe, Racheal | 2021 | Journal of Psychosomatic Research | Prevalence and factors associated with depressive illness in patients with tuberculosis in Mulago hospital Kampala - Uganda: A cross sectional study | Cross sectional study | Uganda |
| Alvarez-Sekely, Magaly | 2023 | Pathogens | Major Depressive Disorder and Pulmonary Tuberculosis Comorbidity Exacerbates Proinflammatory Immune Response - A Preliminary Study | Case control study | Mexico |
| Ambaw, F | 2019 | Global Mental Health | Is depression associated with pathways to care and diagnosis delay in people with tuberculosis in Ethiopia? | Cross sectional study | Ethiopia |
| Ambaw, Fentie | 2017 | BMC Psychiatry | Burden and presentation of depression among newly diagnosed individuals with TB in primacy care settings in Ethiopia | Cross sectional study | Ethiopia |
| Ambaw, Fentie | 2018 | Bulletin of the World Health Organization | Untreated depression and tuberculosis treatment outcomes, quality of life and disability, Ethiopia | Cohort study | Ethiopia |
| Ambaw, Fentie | 2019 | Global Mental Health | Incidence of depression in people with newly diagnosed tuberculosis in Ethiopia: a cohort study | Cohort study | Ethiopia |
| Amole, Taiwo Gboluwaga | 2020 | Nigerian Journal of Medicine | Prevalence and Predictors of Depression among Tuberculosis Patients in Kano, North-West Nigeria | Cross sectional study | Nigeria |
| Amreen, Nadeem | 2021 | Pakistan Journal of Medical & Health Sciences | Pyschiatric Co-Morbidity and its Associated Risk Factors among Tuberculosis Patients | Cross sectional study | Pakistan |
| Aniebue, PN | 2006 | Journal of Nigeria College of Medicine | Prevalence of Depressive Symptoms Amongst Pulmonary Tuberculosis Patients at the University of Nigeria Teaching Hospital, Enugu | Cross sectional study | Nigeria |
| Anjuga Elavarasi, E | 2024 | Indian Journal of Tuberculosis | Depression and stigma experience among patients with tuberculosis in urban and rural settings | Cross sectional study | India |
| Apang, O | 2024 | Indian Journal of Tuberculosis | Screening and management of depression among patients on TB treatment in Papumpare district, Arunachal Pradesh, India | Mixed-Methods | India |
| Assefa, Solomon | 2023 | BMC Psychiatry | Depression, anxiety and their associated factors among patients with tuberculosis attending in Gondar city health facilities, North West Ethiopia | Cross sectional study | Ethiopia |
| Aydin, Ismail Orhan | 2001 | General Hospital Psychiatry | Depression, anxiety comorbidity, and disability in tuberculosis and chronic obstructive pulmonary disease patients: applicability of GHQ-12 | Cross sectional study | Turkey |
| Babar, Masood Uz Zaman | 2023 | Pakistan Journal Medical and Health Sciences | Prevalence and Determinants of Depression among Multi Drug Resistant (MDR) TB Cases Cross-Sectional Observational Study | Cross sectional study | Pakistan |
| Balaji, Arjun L | 2013 | Asian Journal of Psychiatry | Depression in patients with pulmonary tuberculosis in a tertiary care general hospital | Cross sectional study | India |
| Basham, Andrew C | 2021 | Annals of Epidemiology | Tuberculosis-associated depression: a population-based cohort study of people immigrating to British Columbia, Canada, 1985-2015 | Cohort study | Canada |
| Berg, Jill | 2005 | Research in Nursing & Health | Predictors of Screening Results for Depressive Symptoms Among Homeless Adults in Los Angeles With Latent Tuberculosis | Cross sectional study | United States |
| Calligaro, Gregory L | 2021 | Pilot and Feasibility Studies | Brief psychotherapy administered by non-specialised health workers to address risky substance use in patients with multidrug-resistant tuberculosis: a feasibility and acceptability study | Cohort study | South Africa |
| Cere dos Santos, Ana Paula | 2016 | Tuberculosis and Respiratory Diseases | Health-Related Quality of Life, Depression and Anxiety in Hospitalized Patients with Tuberculosis | Cross sectional study | Brazil |
| Chandrashekar, TR | 2012 | Medico-Legal Update | A Study of Psychiatric Morbidity among Patients Suffering from Pulmonary Tuberculosis | Cross sectional study | India |
| Chauhan, Ajay | 2024 | Journal of Family Medicine and Primary Care | Dual burden of TB and mental ill-health: Prevalence and associated factors of anxiety and depression among TB patients in Gujarat | Cross sectional study | India |
| Che Anye, Lionel | 2023 | BJ Psych Open | Depression, anxiety and medication adherence among tuberculosis patients attending treatment centres in Fako Division, Cameroon: cross-sectional study | Cross sectional study | Cameroon |
| Chen, Xu | 2023 | Scientific reports | The role of self-esteem as moderator of the relationship between experienced stigma and anxiety and depression among tuberculosis patients | Cross sectional study | China |
| Cheng, Kao-Chi | 2017 | Frontiers in Psychiatry | Increased Risk of Pulmonary Tuberculosis in Patients with Depression: A Cohort Study in Taiwan | Case control study | Taiwan |
| Chopra, Kamal K | 2024 | Indian Journal of Tuberculosis | Occurrence of anxiety and depression among homeless individuals with pulmonary tuberculosis | Cross sectional study | India |
| Contreras, Carmen | 2024 | Cambridge Prisms: Global Mental Health | Programmatic implementation of depression screening and remote mental health support sessions for persons recently diagnosed with TB in Lima, Peru during the COVID-19 pandemic | Single arm intervention study | Peru |
| Court, Richard | 2021 | International Journal of Infectious Diseases | Neuropsychiatric toxicity and cycloserine concentrations during treatment for multidrug-resistant tuberculosis | Cohort study | South Africa |
| Dan-ni, Zhang | 2024 | Frontiers in Public Health | Prevalence and risk factors of anxiety and depression in patients with multi-drug/rifampicin-resistant tuberculosis | Cross sectional study | China |
| Dar, Shabir Ahmad | 2019 | The Indian Journal of Chest Diseases and Allied Sciences | A Prospective Study on Patterns of Psychiatric Morbidity in Patients with Pulmonary Tuberculosis at a Tertiary Care Hospital in Northern India | Cohort study | India |
| Das, Mrinalini | 2014 | Global Health Action | HIV, multidrug-resistant TB and depressive symptoms: when three conditions collide | Cohort study | India |
| Dasa, Tamirat Tesfaye | 2019 | BMC Psychiatry | Prevalence and associated factors of depression among tuberculosis patients in Eastern Ethiopia | Cross sectional study | Ethiopia |
| de Castro-Silva, Karina M | 2019 | Brazil Journal of Psychiatry | Prevalence of depression among patients with presumptive pulmonary tuberculosis in Rio de Janeiro, Brazil | Cross sectional study | Brazil |
| Devkota, Janardan | 2016 | Janaki Medical College Journal of Medical Science | Health Related Quality of Life, Anxiety and Depression among Tuberculosis Patients in Kathmandu, Nepal | Cross sectional study | Nepal |
| Dixit, Kritika | 2024 | BMC Global and Public Health | Stigma, depression, and quality of life among people with pulmonary tuberculosis diagnosed through active and passive case finding in Nepal: a prospective cohort study | Cohort study | Nepal |
| Djochie, Richard Delali Agbeko | 2025 | PLOS Global Public Health | Factors influencing treatment success in drug-susceptible tuberculosis patients in Ghana: A prospective cohort study | Cohort study | Ghana |
| Dong, Xiaoxin | 2020 | American Journal of Tropical Medicine and Hygiene | Prevalence of Depressive Symptoms and Associated Factors among Internal Migrants with Tuberculosis: A Cross-Sectional Study in China | Cross sectional study | China |
| Duko, Bereket | 2015 | BMC Psychiatry | Prevalence and correlates of depression and anxiety among patients with tuberculosis at WolaitaSodo University Hospital and Sodo Health Center, WolaitaSodo, South Ethiopia, Cross Sectional Study | Cross sectional study | Ethiopia |
| Emami, Habib | 2015 | Tanaffos | Psychological Symptoms Before and After a 14-Day Initial Inpatient Treatment in Tuberculosis Patients Compared with Their Primary Caregivers and Healthy Controls | Cohort study | Iran |
| Fang, Xue-E | 2022 | World Journal of Clinical Cases | Association between depression and malnutrition in pulmonary tuberculosis patients: A cross-sectional study | Cross sectional study | China |
| Fang, Xue-Hui | 2022 | Journal of Multidisciplinary Healthcare | Social Support and Depression Among Pulmonary Tuberculosis Patients in Anhui, China | Cross sectional study | China |
| Febi, Ann Roy | 2021 | ERJ Open Research | Psychological stress and health-related quality of life among tuberculosis patients: a prospective cohort study | Cohort study | India |
| Fleming, MF | 2006 | International Journal of Tuberculosis and Lung Disease | Alcohol and drug use disorders, HIV status and drug resistance in a sample of Russian TB patients | Cross sectional study | Russia |
| Fuady, Ahmad | 2024 | PLOS Global Public Health | Stigma, depression, quality of life, and the need for psychosocial support among people with tuberculosis in Indonesia: A multi-site cross-sectional study | Cross sectional study | Indonesia |
| Furin, JJ | 2001 | International Journal of Tuberculosis and Lung Disease | Occurrence of serious adverse effects in patients receiving community-based therapy for multidrug-resistant tuberculosis | Cohort study | Peru |
| Galea, JT | 2023 | International Journal of Tuberculosis and Lung Disease | Latent TB and depressive symptoms in household contacts of persons with active TB | Cohort study | Peru |
| Galhenage, JS | 2016 | Ceylon Medical Journal | Psychological morbidity and illness perception among patients receiving treatment for tuberculosis in a tertiary care centre in Sri Lanka | Cross sectional study | Sri Lanka |
| Gao, Weiwei | 2021 | American Journal of Translational Research | Influence of anti-tuberculosis drugs plus cycloserine on sputum negative conversion rate, adverse reactions and inflammatory factors in multi-drug resistant tuberculosis | Cohort study | China |
| Garfin, Dana Rose | 2022 | Journal of Health Psychology | Correlates of depression and anxiety among homeless adults with latent tuberculosis infection | Cross sectional study | United States |
| Gong, Yanhong | 2018 | American Journal of Tropical Medicine and Hygiene | Prevalence of Depressive Symptoms and Related Risk Factors among Patients with Tuberculosis in China: A Multistage Cross-Sectional Study | Cross sectional study | China |
| Govender, S | 2009 | South African Family Practice | What are the reasons for patients not adhering to their anti-TB treatment in a South African district hospital? | Case control study | South Africa |
| Hayes-Larson, E | 2017 | International Journal of Tuberculosis and Lung Disease | Depressive symptoms and hazardous/harmful alcohol use are prevalent and correlate with stigma among TB-HIV patients in Lesotho | Cross sectional study | Lesotho |
| Huque, R | 2020 | BMC Public Health | "Death is a better option than being treated like this": a prevalence survey and qualitative study of depression among multi-drug resistant tuberculosis in-patients | Cross sectional study | Bangladesh |
| Husain, Mohammed O | 2008 | Clinical Practice and Epidemiology in Mental Health | The relationship between anxiety, depression and illness perception in tberculosis patients in Pakistan | Cross sectional study | Pakistan |
| Ige, Olusoji Mayowa | 2011 | Mental Health in Family Medicine | Prevalence of depression in tuberculosis patients in comparison with non-tuberculosis family contacts visiting the DOTS clinic in a Nigerian tertiary care hospital and its correlation with disease pattern | Cross sectional study | Nigeria |
| Ilesanmi, Olayinka Stephen | 2020 | West African Journal of Medicine | The Prevalence and Factors Associated with Depression among Patients with Pulmonary Tuberculosis at the Federal medical Center, Owo, Nigeria | Cross sectional study | Nigeria |
| Issa, Baba A | 2009 | Mental Health in Family Medicine | Depression comorbidity among patients with tuberculosis in a university teaching hospital outpatient clinic in Nigeria | Cross sectional study | Nigeria |
| Izhar, MD | 2023 | Periodic Epidemiology Journal | Multiphasic Screening: Clinical Symptoms and Mental Health Patient Tuberculosis in Jambi City, Indonesia | Cross sectional study | Indonesia |
| Jaber, Ammar Ali Saleh | 2016 | PLOS One | Evaluation fo Health-Related Quality of Life among Tuberculosis Patients in Two Cities in Yemen | Cohort study | Yemen |
| Jaffry, Tamkeen Nishat | 2024 | Pakistan Journal of Public Health | Perceived Stigma and Psychological Distress among Tuberculosis Patients: Insights into Psychosocial Challenges at a Healthcare Center | Cross sectional study | Pakistan |
| Jain, Sourabh | 2020 | International Journal of Contemporary Medical Research | Study of Depression among MDR TB Patients - A Crosssectional Observational Study | Cross sectional study | India |
| Jang, Kwang-Sim | 2022 | International Journal of Environmental Research and Public Health | Effects of Simulated Laughter Therapy Using a Breathing Exercise: A Study on Hospitalized Pulmonary Tuberculosis Patients | Quasi-experimental | South Korea |
| Jha, Dharmendra Kumar | 2019 | Perspectives in Clinical Research | Quality of life among HIV-tuberculosis co-infected patients | Cross sectional study | India |
| Jha, Prakash | 2019 | Indian Journal of Mental Health | Prevalence of Depression in Patients with MDR TB | Cross sectional study | India |
| Jones-Patten, Alexandria | 2023 | Clinical Nursing Research | Depression, Anxiety, and Cigarette Smoking Among Patients with Tuberculosis | Cross sectional study | Botswana |
| Judd, FK | 1994 | International Clinical Psychopharmacology | Isoniazid and antidepressants: is there cause for concern? | Case report | Australia |
| Juliasih, Ni Njoman | 2023 | Indonesia Journal of Internal Medicine | Mental Health Status and Its Associated Factors Related to Pulmonary Tuberculosis Patients in Primary Health Care Centre in Surabaya, Indonesia | Cross sectional study | Indonesia |
| Kamble, Bhushan | 2022 | Cureus | Depression Among Patients with Tuberculosis at a Directly Observed Treatment Short-Course (DOTS) Center in Rural Delhi | Cross sectional study | India |
| Kastien-Hillka, Tanja | 2017 | PLOS One | Health-related quality of life in South African patients with pulmonary tuberculosis | Cohort study | South Africa |
| Kaukab, Iram | 2015 | Latin American Journal of Pharmacy | Effect of Pharmacist-Led Patient Education on Management of Depression in Drug Resistance Tuberculosis Patients using Cycloserine. A prospective study | Randomised controlled trial | Pakistan |
| Kehbila, Jules | 2016 | Infectious Diseases of Poverty | Prevalence and correlates of depressive symptoms in adult patients with pulmonary tuberculosis in the Southwest Region of Cameroon | Cross sectional study | Cameroon |
| Khachadourian, Vahe | 2020 | BMC Pulmonary Medicine | People-centred care versus clinic-based DOT for continuation phase TB treatment in Armenia: a cluster randomized trial | Randomised controlled trial | Armenia |
| Kigozi-Male, Gladys | 2024 | Southern African Journal of Infectious Diseases | Possible depression in new tuberculosis patient in the Free State province, South Africa | Cross sectional study | South Africa |
| Kochuieva, Maryna | 2021 | Eureka: Health Sciences | Association of psychological status with first diagnosed pulmonary tuberculosis in men | Cross sectional study | Ukraine |
| Konsaku, Kanjana | 2025 | Clinics and Practice | Factors Associated with Mental Health Problems Among Tuberculosis Patients Attending Tertiary Care Hospitals in the Bangkok Metropolitan Region, Thailand: A Hospital-Based Survey | Cross sectional study | Thailand |
| Koyanagi, Ai | 2017 | BMC Medicine | Depression comorbid with tuberculosis and its impact on health status: cross-sectional analysis of community-based data from 48 low- and middle-income countries | Cross sectional study | 48 LMICs |
| Kumar, Kunal | 2016 | Journal of Family Medicine and Primary Care | A study of prevalence of depression and anxiety in patients suffering from tuberculosis | Cross sectional study | India |
| Kumpuangdee, Siwadon | 2022 | Indian Journal of Tuberculosis | Prevalence od depression and anxiety in pulmonary tuberculosis patients and its association with unsuccessful treatment outcome: A prospective cohort study | Cohort study | Thailand |
| Laxmeshwar, Chinmay | 2022 | PLOS One | Psychiatric comorbidities among patients with complex drug-resistant tuberculosis in Mumbai, India | Cohort study | India |
| Lee, Go Un | 2022 | Journal of Korean Academic Nursing | Effects of Depression, Social Support of Tuberculosis Patients on Self-care | Cross sectional study | South Korea |
| Lee, Li-Yun | 2017 | Journal of Clinical Nursing | Perceived stigma and depression in initially diagnosed pulmonary tuberculosis patients | Cross sectional study | Taiwan |
| Li, Xiucai | 2023 | International Journal of General Medicine | Prevalence and factors associated with depression in patients with diabetes mellitus and pulmonary tuberculosis (DM-PTB): A hospital-based cross-sectional study | Cross sectional study | China |
| Li, Xuhui | 2019 | Basic and Applied Social Psychology | Comprehensive intervention for anxiety and depression among the community elderly with tuberculosis | Randomised controlled trial | China |
| Liu, Kewei | 2021 | Frontiers in Psychiatry | Prevalence and Correlates of Anxiety and Depressive Symptoms in Patients with and without Multi-Drug Resistant Pulmonary Tuberculosis in China | Cross sectional study | China |
| Liu, Kui | 2023 | Psychology Research and Behavior Management | Influencing Factors of Treatment Outcomes Among Patients with Pulmonary Tuberculosis: A structural Equation Model Approach | Cross sectional study | China |
| Liu, Xiangmin | 2022 | Frontiers in Psychiatry | Association between depression or anxiety symptoms and immune-inflammatory characteristics in in-patients with tuberculosis: A cross-sectional study | Cross sectional study | China |
| Liu, Xiangmin | 2025 | Frontiers in Psychiatry | Symptom network analysis of insomnia-depression-anxiety-stigma in tuberculosis patients | Cross sectional study | China |
| Lu, Yanhua | 2021 | American Journal of Translational Research | Effect of targeted nursing intervention plus psychological counseling on quality of life, negative emotions, and complications in patients with extensively drug-resistant tuberculosis | Randomised controlled trial | China |
| Mandal, Ankita | 2023 | Monaldi Archives for Chest Disease | A cross-sectional study to determine the psychological distress among pulmonary tuberculosis patients during COVID-19 pandemic | Cross sectional study | India |
| Manoharam, E | 2001 | Indian Journal of Tuberculosis | Psychiatric morbidity, patients' perspectives of illness and factors associated with poor medication compliance among the tuberculous in Vellore, South India | Cross sectional study | India |
| Maria, Avin | 2020 | Jurnal Ners | The effects of Acceptance and Commitment Therapy (ACT) on Depression in TB-HIV Co-infection patients | Quasi-experimental | Indonesia |
| Marra, Carlo A | 2008 | Chest | Health-related Quality of Life Trajectories Among Adults with Tuberculosis Differences between Latent and Active Infection | Cohort study | Canada |
| Masumoto, S | 2014 | International Journal of Tuberculosis and Lung Disease | Prevalence and associated factors of depressive state among pulmonary tuberculosis patients in Manila, The Philippines | Cross sectional study | The Philippines |
| Mihailov, Oana Mariana | 2024 | Clinics and practice | Clinical Strategies for Identifying Pediatric Patients with Tuberculosis at Risk of Developing Depressive Disorders | Cohort study | Romania |
| Mohammedhussein, Mustefa | 2020 | Neuropsychiatric Disease and Treatment | Prevalence and Associated Factors of Depression and Anxiety Among Patients with Pulmonary Tuberculosis Attending Treatment at Public Health Facilities in Southwest Ethiopia | Cross sectional study | Ethiopia |
| Molebatsi, Keneilwe | 2020 | Global Public Health | Depression and delayed tuberculosis treatment initiation among newly diagnosed patients in Botswana | Cross sectional study | Botswana |
| Molla, Alemayehu | 2019 | Neuropsychiatric Disease and Treatment | Depression and associated factors among patients with tuberculosis in Ethiopia: a cross-sectional study | Cross sectional study | Ethiopia |
| Momanyi, Robina Kerubo | 2024 | PLOS One | Psychiatric morbidity among patients on treatment for tuberculosis at a tertiary referral hospital in Western Kenya | Cross sectional study | Kenya |
| Murugan, Yogesh | 2024 | Cureus | Mental Health Impacts of Multidrug-Resistant Tuberculosis in Patients and Household Contacts: A Mixed Methods Study | Mixed-Methods | India |
| Nabei, Jacob Nignan | 2024 | BMC Infectious Diseases | Predictors of quality of life of TB/HIV co-infected patients in the Northern region of Ghana | Cross sectional study | Ghana |
| Nagpal, Manisha | 2019 | Indian Journal of Public Health Research & Development | Effect of Depression on Treatment Outcome in Multi-Drug Resistant Tuberculosis Patients Under Programmatic Management of Drug resistant TB Services in Amritsar, Punjab | Cross sectional study | India |
| Naidoo, Pamela | 2010 | Social Behavior and Personality | Helplessness, Depression, and Social Support among People being Treated for Tuberculosis in South Africa | Cross sectional study | South Africa |
| Naidu, Thirusha | 2020 | American Journal of Tropical Medicine | Major Depression and Stigma among Individuals with Multidrug-Resistant Tuberculosis in South Africa | Cross sectional study | South Africa |
| Nyamathi, Adeline | 2021 | Nursing Research Online | Effect of a Nurse-led Community Health Worker Intervention on Latent Tuberculosis Medication Completion Among Homeless Adults | Single arm intervention study | United States |
| O'Dwyer, Gisele | 2017 | Health | Illness Experience in a Very Low Income/Poor District of Rio de Janeiro, Brazil: Tuberculosis, Medication Adherence and Common Mental Disorders | Qualitative | Brazil |
| Oh, KH | 2017 | International Journal of Tuberculosis and Lung Disease | Depression and risk of tuberculosis: a nationwide population-based cohort study | Cohort study | South Korea |
| Onu, Desmond Uchechukwu | 2025 | Psychology Health & Medicine | Does perceived social support buffer the impact of psychological distress on health-related quality of life among people living with tuberculosis? | Cross sectional study | Nigeria |
| Panati, Dinesh | 2023 | Clinical Epidemiology and Global Health | Effect of depression on treatment adherence among elderly tuberculosis patients: A prospective interventional study | Cross sectional study | India |
| Pang, Yu | 2022 | Infection and Drug Resistance | High Incidence of Psychiatric Disorders Associated with Cycloserine Treatment of Multidrug-Resistant Tuberculosis Patients: A Cohort Study in Beijing, China | Cohort study | China |
| Paradesi, Ramya Keerthi | 2020 | Journal of Clinical and Diagnostic Research | Prevalence and Associated Factors of Major Depressive Disorder among Pulmonary Tuberculosis Patients | Cross sectional study | India |
| Patel, Anjali | 2024 | Journal of Population Therapeutics & Clinical Pharmacology | Prevalence of Depression and its Correlation Among Registered Tuberculosis Patients | Cross sectional study | India |
| Patel, Jaiminkumar P | 2023 | National Journal of Physiology, Pharmacy and Pharmacoloy | Depression among patients with tuberculosis at a directly observed treatment short-course center in tertiary care hospital of Western Gujarat | Cross sectional study | India |
| Patel, Nirmal | 2024 | Cureus | The Invisible Toll: Unveiling the Prevalence and Predictors of Depression and Anxiety Among Pulmonary Tuberculosis (TB) Patients and Their Households in Gujarat, India | Cross sectional study | India |
| Pathak, Abhishek | 2023 | Medica Innovatica | Clinical correlates, Psychiatric morbidity and Quality of Life in patients of Pulmonary Tuberculosis: A cross-sectional study from a tertiary care hospital in rural North India | Cross sectional study | India |
| Pradhan, Anmoi | 2022 | Frontiers in Psychiatry | Internalized and Perceived Stigma and Depression in Pulmonary Tuberculosis: Do they explain the relationship between drug sensitivity status and adherence? | Cross sectional study | India |
| Purnomo As'hab, Priyo | 2022 | Journal of Public Health Research | The effects of acceptance and commitment therapy on psychosocial impact and adherence of multidrug-resistant tuberculosis patients | Quasi-experimental | Indonesia |
| Putnis, Nina | 2023 | Global Health: Science and Practice | Evaluation of a Depression Intervention in People with HIV and/or TB in Eswatini Primary Care Facilities: Implications for Southern Africa | Mixed-Methods | Eswatini |
| Qiu, Lei | 2019 | American Journal of Tropical Hygiene | Depressive Symptoms Mediate the Associations of Stigma with Medication Adherence and Quality of Life in Tuberculosis Patients in China | Cross sectional study | China |
| Rajeev, Harikrishna | 2022 | National Journal of Community Medicine | Stress, Depression and Coping in Tuberculosis Patients-A Hospital Based Study | Cross sectional study | India |
| Redwood, L | 2021 | International Journal of Tuberculosis and Lung Disease | Depression, stigma and quality of life in people with drug-susceptible TB and drug-resistant TB in Vietnam | Cross sectional study | Vietnam |
| Retnakumar, Charutha | 2022 | Indian Journal of Tuberculosis | Assessment of prevalence of depression and its associated factors among tuberculosis patients in Ernakulam district, Kerala | Cross sectional study | India |
| Rouf, Abdul | 2021 | Journal of Clinical Tuberculosis and Other Mycobacterial Diseases | Depression among Tuberculosis patients and its association with treatment outcomes in district Srinagar | Cohort study | India |
| Rubeen, Rukhsana | 2014 | Acta Medica International | Anxiety and Depression in Tuberculosis Can Create Impact on Quality of Life of Patient | Cross sectional study | Pakistan |
| Ruiz-Grosso, Paulo | 2023 | BMJ Open | Are trajectories of depressive symptoms during the first half of drug-sensitive pulmonary tuberculosis treatment associated with loss to follow-up? A secondary analysis of longitudinal data | Cohort study | Peru |
| Salem, Nidhal Haj | 2016 | Infectious Diseases in Clinical Practice | Miliary Tuberculosis as a Cause of Sudden Death in a Patient Misdiagnosed with Depression | Case report | Tunisia |
| Salnikova, Anna | 2025 | Global Health Action | Depression among people living with tuberculosis and tuberculosis/HIV coinfection in Ukraine: a cross-sectional study | Cross sectional study | Ukraine |
| Salodia, Umang P | 2019 | Indian Journal of Public Health | Depression among Tuberculosis Patients Attending a DOTS Centre in a Rural Area of Delhi: A cross-sectional study | Cross sectional study | India |
| Shahid Basha, S Md | 2024 | International Journal of Pharmaceutical and Clinical Research | Study of Prevalence of Depression Anxiety and Alcohol Abuse in Patients Diagnosed with Pulmonary Tuberculosis on Anti-Tubercular Drugs | Cross sectional study | India |
| Sharma, Deepak | 2024 | Indian Journal of Community Medicine | Prevalence and Predictors of Depression Among Drug-Sensitive Tuberculosis Patients - A Cross-Sectional Study | Cross sectional study | India |
| Sharma, Rashmi | 2022 | Indian Journal of Community Medicine | Prevalence and Determinants of Depression among Multi Drug Resistant (MDR) TB cases registered under National Tuberculosis Elimination Program in Ahmedabad City | Cross sectional study | India |
| Shrestha, P | 2019 | Global Mental Health | Depression among TB patients and associated factors in Kathmandu Valley, Nepal | Cross sectional study | Nepal |
| Shrestha, Sailesh Kumar | 2020 | Journal of Clinical Tuberculosis and Other Mycobacterial Diseases | Prevalence and risk factors of depression in patients with drug-resistant tuberculosis in Nepal: A cross-sectional study | Cross sectional study | Nepal |
| Shyamala, KK | 2018 | Journal of the Association of Physicians of India | Depression: A Neglected Comorbidity in Patients with Tuberculosis | Cross sectional study | India |
| Silva, Luci Meire P | 2024 | Arquivos Brasileiros de Oftalmologia | Assessment of quality of life, psychosocial, and epidemiological aspects in patients diagnosed with tuberculous uveitis | Cross sectional study | Brazil |
| Singh, Lalit | 2015 | Industrial Psychiatry Journal | Psychiatric morbidity in patients of pulmonary tuberculosis-an observational study | Case control study | India |
| Singh, Vijay Kumar | 2017 | International Journal of Basic & Clinical Pharmacology | Effect of Sertraline on biomarker alterations in patients of multidrug resistant tuberculosis with depression: a prospective clinical trial | Single arm intervention study | India |
| Singh, Vijay Kumar | 2023 | Asian Journal of Pharmaceutical and Clinical Research | To evaluate the magnitude of depression in multidrug-resistant tuberculosis patients in a tertiary care teaching hospital | Cross sectional study | India |
| Solanki, Naresh | 2023 | Cureus | A Cross-Sectional Study on the Prevalence of Depression and Associated Factors in Tuberculosis Patients in teh Vidisha Distrcit of Madhya Pradesh, India | Cross sectional study | India |
| Solanki, Sahil R | 2024 | Indian Journal of Tuberculosis | Assessment of anxiety and depression among tuberculosis patients of Ahmedabad, India | Cross sectional study | India |
| Srinivasan, G | 2021 | Indian Journal of Tuberculosis | Prevalence of depression and anxiety among drug resistant tuberculosis: A study in North India | Cross sectional study | India |
| Stanikzai, Muhammad Haroon | 2024 | Indian Journal of Tuberculosis | Depression symptoms among Afghan TB patients: A multi-center study | Cross sectional study | Afghanistan |
| Stanikzai, Muhammad Haroon | 2025 | Indian Journal of Tuberculosis | Anti-TB treatment non-adherence predictors: A multi-center cross-sectional study in Kandahar, Afghanistan | Cross sectional study | Afghanistan |
| Stavrou, P | 2002 | British Journal of Neurosurgery | Frontal lobe tuberculoma masquerading as postnatal depression | Case report | United Kingdom |
| Stoichita, Alexandru | 2021 | Monaldi Archives for Chest Disease | Depression and anxiety symptoms among people with rifampicin-resistant tuberculosis receiving in-patient care in the National Pulmonology Reference Institute in Romania | Cohort study | Romania |
| Sulehri, Muhammad Anwar | 2010 | Annals of Punjab Medical College | Prevalence of Depression Among Tuberculosis Patients | Cross sectional study | Pakistan |
| Sunjaya, Deni Kurniadi | 2022 | Journal of Multidisciplinary Healthcare | The magnitude of Anxiety and Depressive Symptoms Among Tuberculosis Patients in Community-Health Centers Setting During the Peak of COVID-19 Pandemic | Cross sectional study | Indonesia |
| Suryavanshi, N | 2020 | International Journal of Tuberculosis and Lung Disease | Assessment of persistent depression among TB patients | Cohort study | India |
| Susanto, Tirta Darmawan | 2023 | Dialogues in Health | Anxiety and depression level of patients with multidrug-resistant tuberculosis (MDR-TB) in two hospitals in Banten province, Indonesia | Cross sectional study | Indonesia |
| Tariq, Amna | 2018 | Pakistan Journal of Medical & Health Sciences | Frequency of Depression in Tuberculosis Patients and its Association with Various Sociodemographic Factors | Cross sectional study | Pakistan |
| Theingi, Phyo | 2021 | Tropical Medicine and International Health | Depression and its associated factors among people with multidrug-resistant tuberculosis in Myanmar | Cross sectional study | Myanmar |
| Thiruvalluvan, E | 2020 | SAARC Journal of Tuberculosis, Lung Diseases & HIV/AIDS | Intervention Strategies to Mitigate Psychosocial Challenges and Improve the Quality of Life of MDR-TB Patients - An evaluation study | Single arm intervention study | India |
| Thungana, Yanga | 2022 | BMJ Open | Comorbidity of mental ill-health in tuberculosis patients under treatment in a rural province of South Africa: a cross-sectional survey | Cross sectional study | South Africa |
| Timilsina, S | 2014 | SAARC Journal of Tuberculosis, Lung Disease & HIV/AIDS | Assessing Quality of Life and Depression Among People Living with HIV/AIDS and TB-HIV Coinfection in Kathmandu, Nepal | Cross sectional study | Nepal |
| Tomita, Andrew | 2019 | Social Psychiatry and Psychiatric Epidemiology | Major depression and household food insecurity among individuals with multidrug-resistant tuberculosis (MDR-TB) in South Africa | Cross sectional study | South Africa |
| Tornheim, JA | 2022 | European Respiratory Jounral | Cycloserine did not increase depression incidence or severity at standard dosing for multidrug-resistant tuberculosis | Cohort study | India |
| Ugarte-Gil, Cesar | 2013 | PLOS One | Association of Major Depressive Episode with Negative Outcomes of Tuberculosis Treatment | Cohort study | Peru |
| Vaidya, Shrutangi | 2024 | Indian Journal of Tuberculosis | Change in psychological parameters and quality of life among individuals with pulmonary and extrapulmonary tuberculosis following the intensive phase of therapy: A longitudinal observational study from central India | Cohort study | India |
| van den Heuvel, Leigh | 2013 | AIDS Care | Frequency and correlates of anxiety and mood disorders among TB- and HIV-infected Zambians | Cross sectional study | Zambia |
| Vega, P | 2004 | International Journal of Tuberculosis and Lung Disease | Psychiatric issues in the management of patients with multidrug-resistant tuberculosis | Case series | Peru |
| Vishwakarma, Deepak | 2020 | Indian Journal of Public Health Research & Development | Prevalence and Determinants of Depression among Patients with Tuberculosis in Municipal Corporation of Sagar District in the State of Madhya Pradesh, India | Cross sectional study | India |
| Walker, Ian F | 2018 | PLOS One | Implementation of a psychosocial support package for people receiving treatment for multidrug-resistant tuberculosis in Nepal: A feasibility and acceptability study | Single arm intervention study | Nepal |
| Walker, IF | 2018 | International Journal of Tuberculosis and Lung Disease | Depression among multidrug-resistant tuberculosis patients in Punjab, Pakistan: a large cross-sectional study | Cross sectional study | Pakistan |
| Walker, IF | 2019 | Public Health Action | Depression and anxiety in patients with multidrug-resistant tuberculosis in Nepal: an observational study | Cohort study | Nepal |
| Wang, Qiao | 2020 | Public Health Nutrition | The association of household food insecurity and HIV infection with common mental disorders among newly diagnosed tuberculosis patients in Botswana | Cross sectional study | Botswana |
| Wang, Xiao-bo | 2018 | Frontiers in Psychiatry | A survey of Anxiety and Depressive Symptoms in Pulmonary Tuberculosis Patients wiwht and without Tracheobronchial Tuberculsis | Cross sectional study | China |
| Westaway, Margaret S | 1992 | Social Science & Medicine | Depression and Self-esteem: Rapid Screening for Depression in Black, Low Literacy, Hospitalized Tuberculosis Patients | Cross sectional study | South Africa |
| Xavier, Paulo Benvinda | 2015 | Africa Health Sciences | Emotional distress in Angolan patients with several types of tuberculosis | Cross sectional study | Angola |
| Xie, Lei | 2023 | Medicine Open | Development and evaluation of an early rehabilitation nursing program for patients with pulmonary tuberculosis | Randomised controlled trial | China |
| Yadav, Prashant | 2024 | Annals of African Medicine | Assessment of Psychiatric ILlness among Pulmonary Tuberculosis Patients at a Tertiary Care Center in India | Cross sectional study | India |
| Yan, Shijiao | 2017 | American Journal of Tropical Medicine and Hygiene | Nonadherence to Antituberculosis Medications: The Impact of Stigma and Depressive Symptoms | Cross sectional study | China |
| Yen, Yung-Feng | 2015 | Journal of Clinical Psychiatry | Association of Pulmonary Tuberculosis and Ethambutol with Incident Depressive Disorder: A Nationwide, Population-Based Cohort Study | Case control study | Taiwan |
| Yilmaz, Ayla | 2016 | Acta Paulista de Enfermagem | Assessment of anxiety, depression, loneliness and stigmatization in patients with Tuberculosis | Cross sectional study | Turkey |
| Ying, Tiong Chong | 2020 | Sains Malaysiana | Prevalence of Depression and Associated Factors Among Tuberculosis Patients in Primary Care in the District of Kuching, Sarawak | Cross sectional study | Malaysia |
| Yohannes, Kalkidan | 2020 | BMC Public Health | Prevalence of depressive symptoms and associated factors among patients with tuberculosis attending public helaht institutions in Gede'o zone, South Ethiopia | Cross sectional study | Ethiopia |
| Yousaf, Anoosha | 2022 | Med Forum | Prevalence of Depression and Anxiety among Patients of Pulmonary Tuberculosis in Outpatient TB clinic of Bahawalpur Medical and Dental College, Bahawalpur | Cross sectional study | Pakistan |
| Yuan, Wei | 2024 | Alternative Therapies | Integrated Nursing and Psychological Intervention for Tuberculosis Complicated by Lung Cancer: Clinical Efficacy | Randomised controlled trial | China |
| Zainuddin, Andi Alfian | 2020 | Indian Journal of Community Medicine | Depression among Patients with Pulmonary Tuberculosis at the State Hospital of Makassar | Cross sectional study | Indonesia |
| Zhang, Li | 2018 | Neuropsychiatric Disease and Treatment | Vitamin D3 as adjunctive therapy in the treatment of depression in tuberculosis patients: a short-term pilot randomized double-blind controlled study | Randomised controlled trial | China |
| Zuo, Xiaowei | 2022 | BMC Public Health | Cognitive-behavioral therapy on psychological stress and quality of life in subjects with pulmonary tuberculosis: a community-based cluster randomized controlled trial | Randomised controlled trial | China |
